# Computationally designed lattices with tuned properties for tissue engineering using 3D printing

**DOI:** 10.1371/journal.pone.0182902

**Published:** 2017-08-10

**Authors:** Paul F. Egan, Veronica C. Gonella, Max Engensperger, Stephen J. Ferguson, Kristina Shea

**Affiliations:** 1 Department of Health Sciences and Technology, Institute of Biomechanics, Swiss Federal Institute of Technology, Zurich, Switzerland; 2 Biomedical Computer Science and Mechatronics, UMIT The Health and Life Sciences University, Hall in Tirol, Austria; 3 Department of Mechanical and Process Engineering, Swiss Federal Institute of Technology, Zurich, Switzerland; University of Zaragoza, SPAIN

## Abstract

Tissue scaffolds provide structural support while facilitating tissue growth, but are challenging to design due to diverse property trade-offs. Here, a computational approach was developed for modeling scaffolds with lattice structures of eight different topologies and assessing properties relevant to bone tissue engineering applications. Evaluated properties include porosity, pore size, surface-volume ratio, elastic modulus, shear modulus, and permeability. Lattice topologies were generated by patterning beam-based unit cells, with design parameters for beam diameter and unit cell length. Finite element simulations were conducted for each topology and quantified how elastic modulus and shear modulus scale with porosity, and how permeability scales with porosity cubed over surface-volume ratio squared. Lattices were compared with controlled properties related to porosity and pore size. Relative comparisons suggest that lattice topology leads to specializations in achievable properties. For instance, Cube topologies tend to have high elastic and low shear moduli while Octet topologies have high shear moduli and surface-volume ratios but low permeability. The developed method was utilized to analyze property trade-offs as beam diameter was altered for a given topology, and used to prototype a 3D printed lattice embedded in an interbody cage for spinal fusion treatments. Findings provide a basis for modeling and understanding relative differences among beam-based lattices designed to facilitate bone tissue growth.

## Introduction

Tissue scaffolds provide *in vivo* mechanical support while facilitating targeted tissue growth, and are commonly used as external intervention in regenerative medicine for supporting bone growth [1]. Challenges remain in developing optimized tissue scaffolds, due to the complexity in tuning a scaffold’s form for both mechanical integrity and biological support [2]. An emerging strategy is the use of additively manufactured beam-based lattices to construct scaffolds with favorable mechanical properties [3–6]. Stretch-dominated lattices with beams deforming axially under load can achieve a higher mechanical efficiency compared to bending dominated structures of similar density, such as foams [7–10]. Recent studies have demonstrated the capacity for tissue growth on beam-based lattices that inform computational approaches for scaffold design [11–13]. Lattice properties linked to scaffold performance include tissue growth related morphological properties of porosity, pore size, and surface area [14], mechanically related properties of elastic modulus and shear modulus [15–17], and properties related to nutrient transport such as permeability [18,19]. Our aim is to computationally model lattices with designed properties suitable for tissue engineering, and provide a basis for comparing lattices as porous structures for bone growth, using a spinal interbody fusion cage application to inform lattice design decisions.

**Fig 1:**
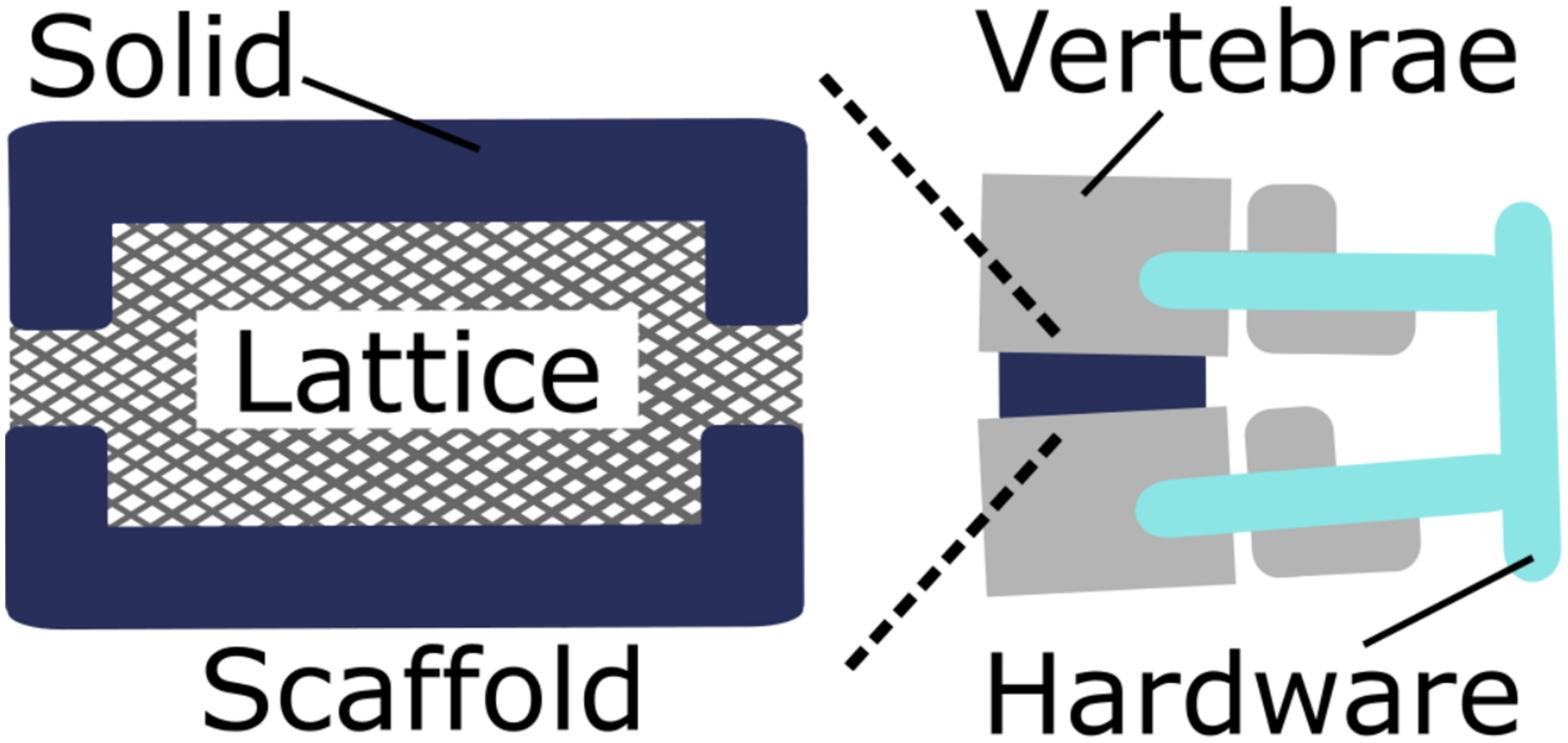
Spinal fusion system schematic. A lattice with a solid shell acts as a tissue scaffold between adjacent vertebrae to facilitate bone fusion.

Spinal interbody fusion cages are scaffolds inserted in the body after complete or partial surgical removal of an intervertebral disc. Cages are designed to bear mechanical load while providing a biological niche for vertebral fusion [20–23]. Once a cage is inserted in the body, it bears spinal loads and provides a scaffolding for cells to populate and begin forming bone that eventually creates one solid bone fusion bridging the adjacent vertebrae. Cage designs often contain a solid shell surrounding a porous medium, such as a lattice. The cage case study illustrates the flexibility in mechanical requirements for the lattice, since other aspects of the system additionally carry load. Therefore, lattice geometry may be balanced for an optimized trade-off between mechanical and biological requirements. Hardware including pedicle screws are inserted in adjacent vertebrae and [24] provide additional mechanical support (Fig 1).

Mechanical loads are distributed among the porous lattice, solid shell, and hardware during spinal extension, flexion, bending, and rotation [20,21]. Due to load distribution, the lattice only bears a portion of the 0.1*MPa* to 2*MPa* compressive load previously experienced by the removed intervertebral disc [25]. Cage designs include single and dual cage systems with overall dimensions typically between 10*mm* and 20*mm* [26]. Cages may be constructed with biocompatible materials that are either absorbed by the body, such as ceramics resulting in the dissolution of the scaffold, or remain in the body after bone fusion, such as metals. Spinal cage porosity, which is the fraction of void volume in comparison to total nominal volume, typically ranges from 0.5 to 0.9. Higher porosity is desirable since it enables greater tissue growth volume and nutrient transport efficiency.

Porosity is inversely proportional to a lattice’s relative density, so mechanical properties that scale with relative density, such as elastic and shear modulus, are reduced as porosity increases [27]. The scaling of mechanical properties with porosity also depends on a lattice’s topology [7]. For unidirectional loads, such as compressive spinal loading, lattices with a greater proportion of beams aligned with the loading direction have higher elastic moduli, while those with a higher proportion of diagonally aligned beams have higher shear moduli [28–30]. The appropriate tuning of elastic and shear moduli depends on multiple factors, such as the material choice for the scaffold and proportion of load carried by the pedicle screws. Common biocompatible materials used for cages include metals, ceramics, and polymers [2]. The elastic and shear moduli determine a scaffold’s deformation under load that may influence mechanotransduction of growing tissue, such as stress shielding that occurs when a scaffold’s elastic modulus is greater than surrounding bone [29].

Properties related to biological growth and nutrient transport including pore size, surface-volume ratio (i.e. interior lattice surface area divided by nominal volume), and permeability also depend on lattice topology. Pore size refers to the size of void cavities throughout a scaffold, with smaller pores (200μm) being desirable since they enable growing tissue to fill cavities faster, although larger pores (800μm) are required for vascularization and nutrient transport [31]. Smaller pores promote higher surface-volume ratio necessary for initial cell attachment. Permeability is a measure of fluid flow through a porous material, with higher permeability ensuring nutrients are provided and waste is removed [32]. Permeability has been empirically demonstrated for bone [33] and stochastic foam scaffolds [10] to scale with porosity cubed over surface-volume ratio squared, referred to as the Kozeny-Carmen relation [34–36].

When considering mechanical and biological properties for tuning lattices such as tissue scaffolds, there is a complex coupling of properties based on topology, porosity, and pore size [37–39]. Over the past twenty years, a considerable amount of research has focused on determining how scaffold geometry is coupled to these properties and thus determines a scaffold’s mechanical and biological function [40–42]. A key consideration is how tissues grow in response to mechanical stimuli that are related to scaffold properties [42–45], in addition to fluid shear stress [40,46]. Additionally, the geometry of a scaffold can influence tissue growth rates, since tissue generation is influenced by local curvature [47]. Due to the complexity of factors that influence tissue growth, studies typically concentrate on in-depth analysis of how tissue growth is related to simple geometric properties of the scaffold [43,44], or how optimization of topology may occur in relation to key constraints [48]. There is a need for new studies that demonstrate relative trade-offs in scaffold design optimization, to achieve favorable properties, when considering many factors simultaneously and complex topologies, such as beam-based lattices.

Emerging trends in 3D printing processes are now enabling the design and fabrication of high-strength, open porous scaffolds constructed from beam elements [3,5,28], that may provide favorable property trade-offs when considering mechanical and biological factors, since they retain high relative strength for highly porous structures [2]. Recent studies have begun defining the geometries of diverse topologies for tissue engineering from beam-based designs [49], however, much work is still required to analyze and compare lattices of varied topologies to assess the configuration of scaffolds with diverse properties. Due to the large number of trade-offs and analyses concerning scaffold performance, studies typically concentrate on controlling and analyzing a small subset of properties, such as scaffold mechanics using finite element simulations [29] or permeability using computational fluid dynamics [50].

**Fig 2:**
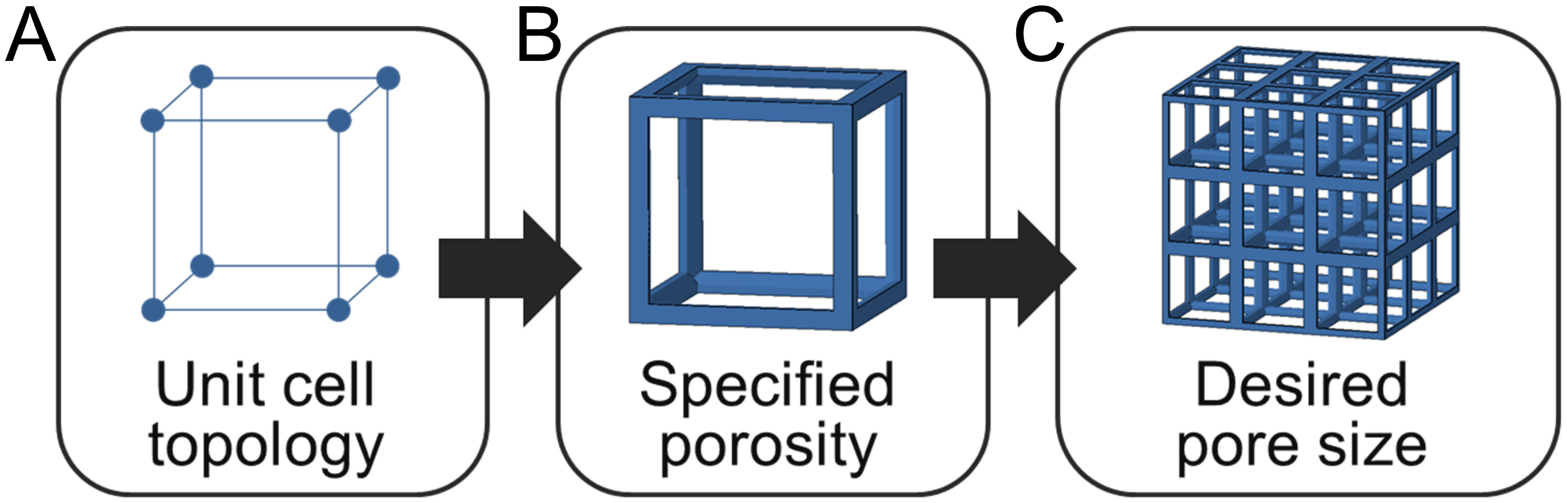
Lattice design approach for tissue scaffolds. (a) A defined topology is used to construct unit cells with (b) a specified porosity for (c) generating a lattice structure with desired pore sizes.

There is a need for developing effective design approaches for configuring lattices with favorable property trade-offs [51–53], particularly with the aid of computational tools that incorporate aspects for assessing both mechanical and biological aspects of scaffolds [54]. Lattice design is often challenging even from a purely mechanical perspective [55,56], and is often conducted computationally. Computational approaches have enabled the comparison of spinal interbody cage properties designed as solid structures with spherical pores [57,58], that can inform the basis of a beam-based lattice modeling approach. Parametric approaches may aid in designing lattices for scaffold applications by taking advantage of parameter coupling and scaling relationships, which has been a successful approach for complex biomechanical systems design [59,60]. In this paper, we develop a computational approach that enables controlled comparisons of diverse beam-based lattices by assuming an initial unit cell topology, specifying porosity, and generating lattices with a desired pore size by patterning identical unit cells (Fig 2).

The unit cell topology is initially specified in Fig 2A to simplify the complex design space by utilizing the common approach of selecting topologies from a library of unit cells, rather than including topological changes as a design parameter [49]. Initial topologies provide a starting point for tuning scaffold properties by altering parameters of beam diameter and unit cell length. Unit cell topology provides a basis for determining beam alignment with loading directions, whether a unit cell is stretch or bending dominated, and the relationships of pore size with beam diameter and unit cell length. Such an approach builds upon previous studies that have characterized the geometrical trade-offs of lattice libraries [49], while incorporating new assessments of properties related to the mechanical and biological function of the scaffold.

Once a topology is chosen, porosity is determined (Fig 2B) based on the ratio of beam diameter to unit cell length that defines a lattice’s relative density [5]. Mechanical properties of elastic modulus and shear modulus remain static for a specified porosity regardless of the configured pore size since the unit cell maintains a fixed relative density when it is rescaled to generate a lattice in Fig 2C with appropriate pore sizes. Once unit cells are rescaled, the resulting element diameter and unit cell length are used to calculate surface-volume ratio and permeability. These steps provide a basis for rapid configuration of a lattice’s structure for multiple controlled properties simultaneously, thereby facilitating the design and assessment of scaffold libraries for diverse properties, such as spinal interbody cage applications.

The goal of this paper is to develop a computational approach for analyzing lattice properties relevant to bone tissue engineering and compare the relative differences among lattices of varied topologies. Computer-aided design software is used with finite element simulations to generate lattices and quantify property relationships. Significant contributions include the development of a design approach for tailoring 3D printed beam-based lattices with controlled properties, use of multiple types of analyses to characterize trends in scaffold properties related to topology, and comparing relative differences among designed lattices when considering numerous properties simultaneously. Comparisons are conducted in the context of bone tissue engineering, particularly for spinal fusion applications, with a final spinal interbody cage prototype defined by selecting a suitable topology based on its relative property tunings, and 3D printed as a proof of concept. Outcomes provide a basis for determining relative differences among achievable lattice properties for designs relevant to regenerative medicine approaches for bone tissue engineering.

## Methods

### Unit cell library

**Fig 3:**
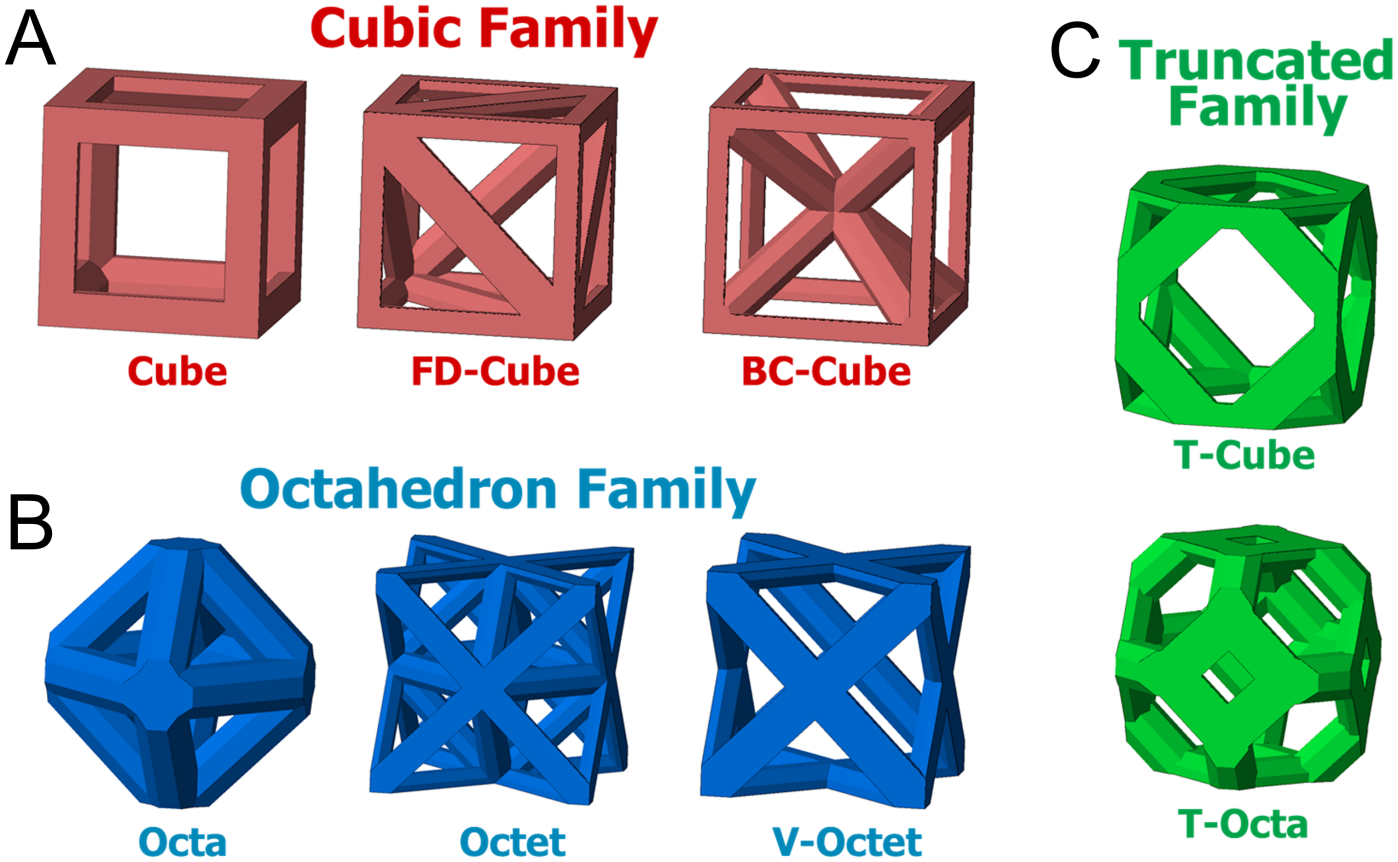
Unit cell families. Unit cells are grouped in (A) Cubic, (B) Octahedron, and (C) Truncated families based on their topology. Illustrated unit cells have porosity *P* = 0.8.

Unit cells were generated as cubic volumes with edges of unit cell length *L*_*c*_. All beams have octagonal cross-sections and beam diameter ø that are centered on beam connections. The material portion of beams that extends beyond the cubic volume were removed to ensure no overlaps occur when unit cells are patterned [61]. A library of eight unit cells was created by generating topologies of varied beam organizations within the cubic volume. Unit cells were grouped within three families that share topological features. The Cubic family (Fig 3A) has beams along each cubic volume edge and a variable number of diagonal beams, the Octahedron family (Fig 3B) has no beams along cubic edges and only has diagonal beams [29], and the Truncated family has unit cells that introduce additional beams by altering the Cube or Octahedron unit cells (Fig 3C).

The Cube cell has beams only along each edge of the cubic volume. The Face Diagonal Cube (FD-Cube) cell retains the Cube cell beams and adds a diagonal beam on each face. The Body-Centered Cube (BC-Cube) cell also retains the Cube cell beams and adds a beam from each corner to the cubic volume center [61].

The Octahedron (Octa) cell has beams that begin and end at the center of each cubic volume face. The Octa cell has a similar geometry to the Cube cell but beams are diagonal and shorter in relation to the cubic volume. The Octet (Octet) cell retains the same beams as the Octa cell, but adds additional beams to form a tetrahedron in each cubic volume corner [27]. The Void Octet (V-Octet) cell contains the beams of the Octet cell not present in the Octa cell.

Truncated unit cells replace connection points of the Cube and Octa cells with beam combinations that form triangular or square planar interconnectivity pores. The Truncated Cube (T-Cube) cell introduces triangular shapes at each corner of the Cube cell [49]. Each triangular shape has a beam that begins and ends at a distance of 0.42 *L*_*c*_ from the corner of the cubic volume along each edge; this distance ensures that access to pores in each corner remain accessible for porosities of 0.6 and higher. The Truncated Octahedron (T-Octa) cell was formed by introducing a square shape for each Octa unit cell connection [9].

### Morphological model

Lattices consisted of a cubic volume of unit cells for a single topology, with the same number of unit cells in all directions with uniform beam diameters. Adjacent FD-Cube unit cells were mirrored to ensure adjacent unit cells share diagonal beams. Porosity *P* was calculated by dividing a lattice’s void volume by its nominal volume. Surface-volume ratio *S*/*V* was calculated by dividing a lattice’s inner surface area by its nominal volume. The inner surface area is the exposed surface area of a lattice minus the surface area of each face. Porosity and surface-volume ratio were found using Abaqus software.

**Fig 4:**
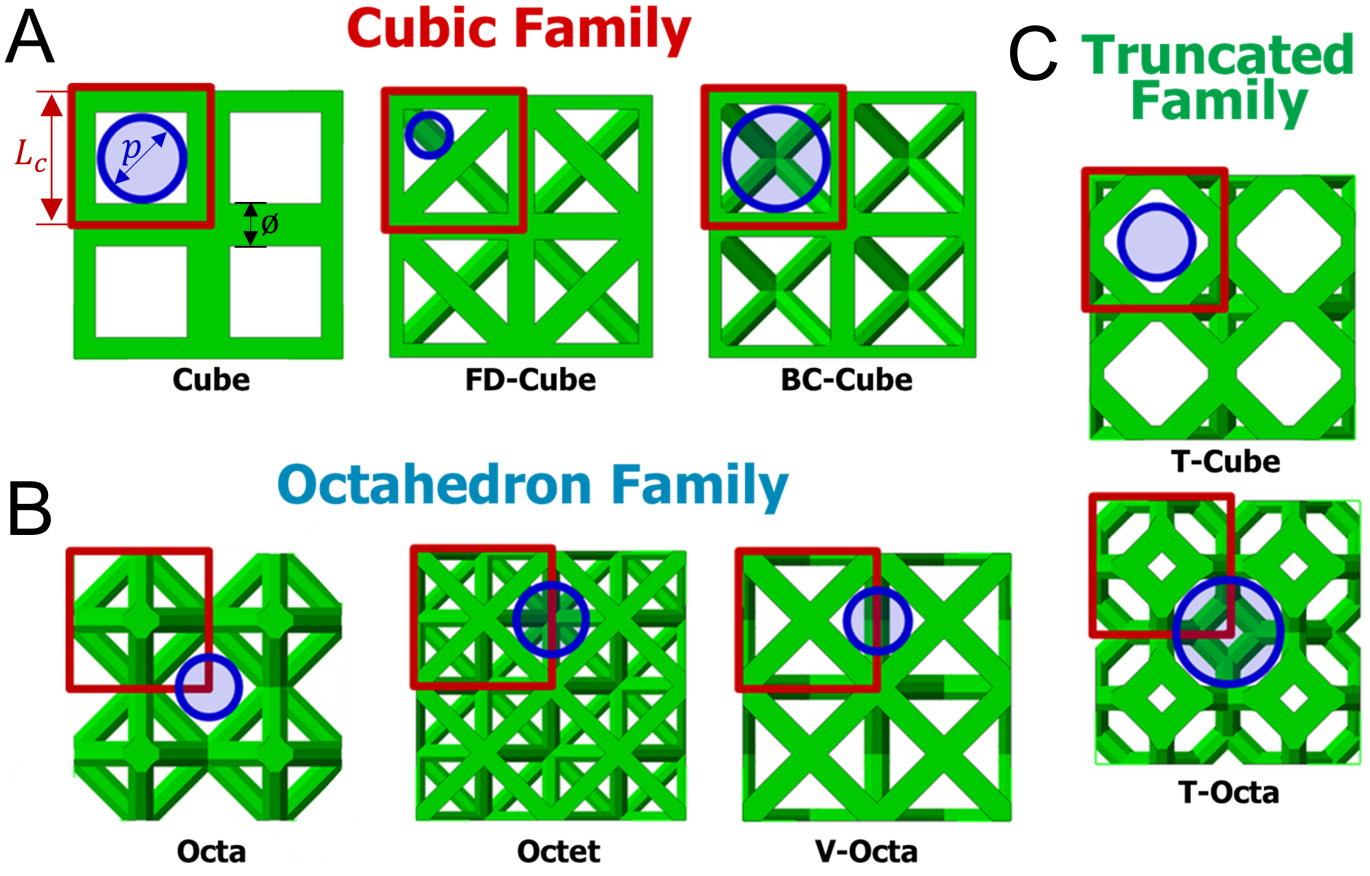
Defined pore sizes. Lattices with porosity *P* = 0.8 have overlaid squares indicating unit cell boundaries with beam diameters ø, unit cell length *L*_*c*_, and circles indicating defined pores with diameter *p*.

Pore size *p* was defined as the diameter of the largest circular area aligned with a lattice face, is surrounded on the majority of its perimeter by in-plane beams, and has no intersections with beams [28]. Defined pores are interconnections of larger three-dimensional porous voids in each lattice, and chosen as the primary comparative metric since interconnectivity pores typically fill prior to larger three dimensional spaces in beam-based scaffolds [12]. Depending on the topology, defined pores are on the face of each unit cell (Cube, FD-Cube, BC-Cube, and T-Cube), the intersection of adjacent unit cells (Octet and V-Octet), or the intersection of four unit cells (Octa and T-Octa) as shown in Fig 4.

Pore sizes were calculated with unit cell length *L*_*c*_ and beam diameter ø parameters; for the Cube topology pore size is calculated by subtracting the length of the element diameter from the length of the unit cell as

$$p=L_{c}-ø$$ (1)

The BC-Cube and T-Octa cells have pore size *p* = *L*_*c*_−*ø* as found in Eq 1, while the Octa, Octet, and V-Octa cells have $p=\frac{\sqrt{2}}{2}(L_{c}-ø)$, and the T-Cube cell has *p* = 0.84 *L*_*c*_−ø based on differences in their geometries. The FD-Cube cell has $p=\sqrt{2}l\;\sqrt{\frac{2l-\sqrt{2}l}{2l+\sqrt{2}l}}$, with $l=\left(L_{c}-ø\right)-\frac{\sqrt{2}ø}{2}$. The FD-Cube cell has a more complicated geometrical relationship since its pore resides in a triangular shape [28].

### Mechanical simulations

Elastic modulus and shear modulus were calculated using a beam analysis [2,29,62]. Each beam was composed of three finite elements with Abaqus software and 125 unit cells were patterned to fill a cubic volume. Beams entirely on a lattice face had their cross-sectional area divided by half, while beams along each lattice edge had their area divided by four; these adjustments correct for the cutoff of beams that extend beyond the cubic volume of a unit cell. An elastic modulus and shear modulus of 1*MPa* was assumed for the base material, and facilitates relative comparisons that are material independent [27]. For all simulations, the mechanical behavior of each beam is approximated with the Euler-Bernouli beam theorem, with quadratic shape functions used and element sizes set to one third of each beam’s length. Therefore, the total number of elements in each simulation is proportional to the number of beams.

The relative elastic modulus *Er* was found by applying a unidirectional displacement *δ* = 0.01 *L* to all nodes on one lattice face, where *L* is the lattice length. Nodes on the opposite face were fixed in the displacement direction. Additional displacement constraints were applied to two corner nodes to constrain rotation, and included one pin and one unidirectional displacement constraint. The reaction force *F* was used to calculate the relative elastic modulus as

$$E_{r}=\frac{F\cdot L}{A\cdot \delta }$$ (2)

where *A* is the area of a lattice face [29].

The relative shear modulus

$$G_{r}=\frac{F\cdot L}{A\cdot \delta }$$ (3)

was found by applying unidirectional displacement constraints of *δ* = 0.01 *L* on adjacent faces towards their common edge. Opposite faces were fixed to not displace in the same direction as their opposing face’s displacement. Constraints were applied so the lattice does not rotate out of plane and a pin was added in one corner. The reaction force was found on each face and averaged to calculate the shear modulus. Boundary conditions for the elastic and shear modulus were generated to represent a general case for determining lattice material properties.

### Fluid flow simulation

Permeability *k* was determined with ANSYS fluent computational fluid dynamics software that simulated unidirectional fluid flow through a lattice. Walls were placed around four sides of a lattice consisting of 27 unit cells that represent a flow channel [10,18,63]. Navier-Stokes equations of continuity and momentum were solved with the finite volume method. The fluid was modeled as incompressible water with a viscosity of *v* = 0.001*Pa s* and 1000*kg*/*m*^3^ density [19]. Boundary conditions included an inlet flow of 0.001*m*/*s*, null outlet pressure, and no-slip conditions. The boundary conditions ensured that fluid flow was laminar with a Reynolds number lower than ten.

Permeability was found using Darcy’s equation

$$k=\frac{Q\nu L}{\Delta pA}$$ (4)

with *Q* as the volumetric flow rate, *L* as the lattice length, *A* as the lattice cross-sectional area, and *Δp* as the pressure gradient across the fluid domain [10]. The outlet velocity and pressure in the center of lattice were used to determine the permeability [64]. The calculated permeability is representative of an intrinsic permeability that is independent of the number of unit cells.

## Results

### Morphological properties

**Fig 5:**
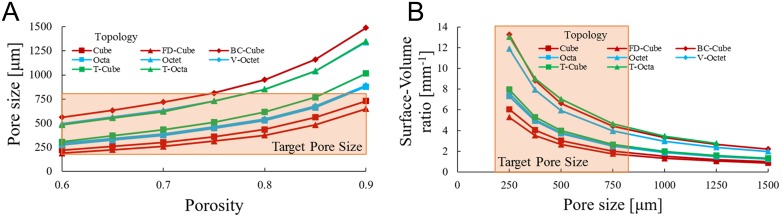
Morphological trends. (a) Pore size *p* plotted for porosity *P* when beam diameter ø = 200μ*m* and (b) surface-volume ratio *S*/*V* plotted as pore size increases from 250μ*m* to 1500μ*m* for lattices with *P* = 0.8 for all topologies.

Trends among lattice porosity *P*, pore size *p*, and surface-volume ratio *S*/*V* were analyzed by altering beam diameter ø and unit cell length *L*_*c*_ to facilitate topology comparisons with controlled porosity. In Fig 5A pore size was increased by holding beam diameter at ø = 200*μm* and increasing unit cell length for a porosity range of *P* = 0.6 to *P* = 0.9.

Fig 5A results show pore size increasing exponentially with porosity, since unit cells may become increasingly larger but porosity may not exceed unity. The scaling results in a greater relative difference in pore sizes among topologies as porosity increases, particularly above *P* = 0.8. The BC-Cube lattice has the largest pore size at a given porosity while FD-Cube has the smallest. The relative difference in pore size among topologies depends on the ratio of pore size to unit cell length (large for BC-Cube, small for FD-Cube) and the diameter to length ratio of beams (large for Cube, small for BC-Cube) at a given porosity.

For a given porosity, beam diameter and unit cell length are tunable to achieve any pore size for a unit cell, although there are practical constraints. The smallest achievable pore size depends on the minimum manufacturable beam diameter, which is about 200μ*m* for titanium scaffolds constructed with selective laser sintering [28] and smaller for stereolithography processes with polymers [65]. To facilitate bone tissue growth, scaffolds require pores greater than 50μ*m* for vascularization and mineralization [14] and less than 1000μ*m* since larger pores slow curvature-dependent tissue growth rates [12]. Typically, pore sizes from 200μ*m* to 800μ*m* are considered optimal for bone growth, and are indicated as the “Target Pore Size” in Fig 5 [1].

Alterations in pore size while porosity was held constant were used to find variations in a lattice’s surface-volume ratio. Surface-volume ratio is plotted in Fig 5B by holding porosity at *P* = 0.8 while tuning beam diameter and unit cell length to generate topologies with pore sizes between *p* = 250μ*m* and *p* = 1500μ*m*. Surface-volume ratio increases exponentially as pore size decreases for all topologies, since pore size must remain positive while surface-volume ratio is unbounded. Lattices with more beams per unit cell such as the BC-Cube, Octet, and T-Octa cells tend to have the greatest surface-volume ratio for a given pore size while the Cube and FD-Cube cells have the lowest.

### Mechanical properties

**Fig 6:**
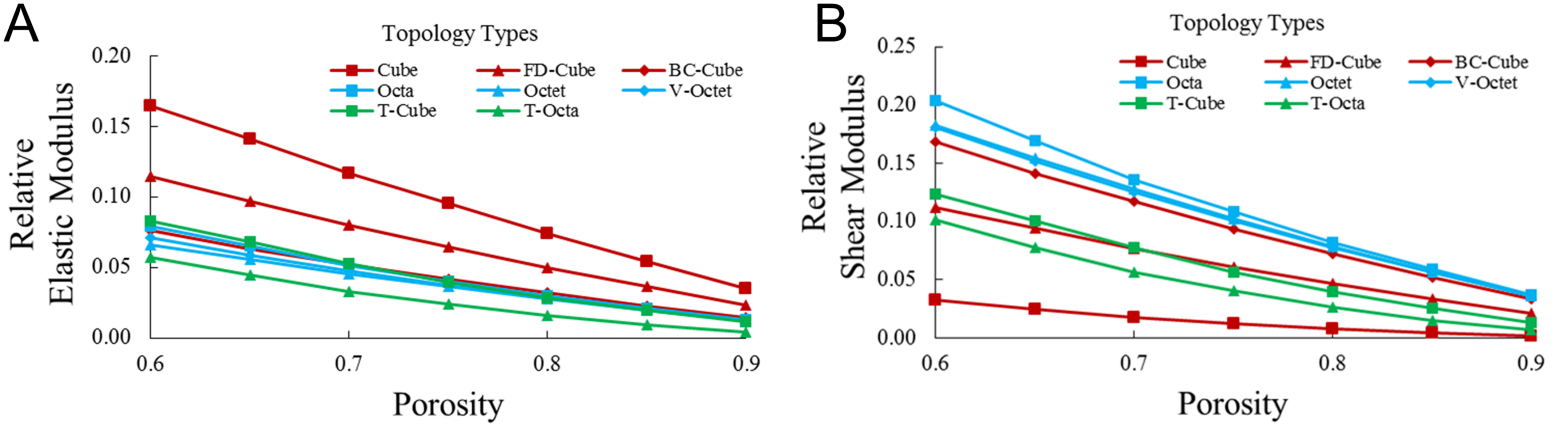
Mechanical trends. (a) Relative elastic modulus *E*_*r*_ and (b) relative shear modulus *G*_*r*_ for porosity *P* for all topologies.

Mechanical properties were simulated by generating lattices with beam diameter ø = 200*μm* and increasing unit cell length to achieve porosities between *P* = 0.6 and *P* = 0.9. Simulation results for relative elastic modulus *E*_*r*_ and relative shear modulus *G*_*r*_ are plotted in Fig 6A and 6B, respectively. Relative elastic moduli were found by dividing the modulus found from finite element analysis by the modulus of the base material of the simulation. Findings should remain independent of material choice for small displacements.

As porosity increases, relative elastic modulus and relative shear modulus decrease for all topologies. In Fig 6A, the Cube topology has the highest relative elastic modulus (*E*_*r*_ ≈ 0.16 at *P* = 0.6) since it has the highest proportion of beams aligned with the loading direction. The FD-Cube topology has the second highest relative elastic modulus (*E*_*r*_ ≈ 0.12 at *P* = 0.6), while all other topologies have similar relative elastic moduli (*E*_*r*_ ≈ 0.065–0.08 at *P* = 0.6) except the T-Octa (*E*_*r*_ ≈ 0.055 at *P* = 0.6). The T-Octa’s relatively low elastic modulus is possibly based on it being a bending dominated topology that is generally considered well-suited for applications necessitating energy absorption, rather than stiffness [9].

In Fig 6B, the Cube topology has the lowest relative shearing modulus (*G*_*r*_ ≈ 0.04 at *P* = 0.6). Octahedron family topologies have high relative shear moduli, with the Octa topology having a slightly higher relative shear modulus (*G*_*r*_ ≈ 0.2 at *P* = 0.6) than the Octet and V-Octet topologies (*G*_*r*_ ≈ 0.17 at *P* = 0.6). The BC-Cube topology has a slightly lower relative shear modulus (*G*_*r*_ ≈ 0.16 at *P* = 0.6) while the remaining topologies have similar relative shear moduli (*G*_*r*_ ≈ 0.10–0.12 at *P* = 0.6).

### Permeability properties

**Fig 7:**
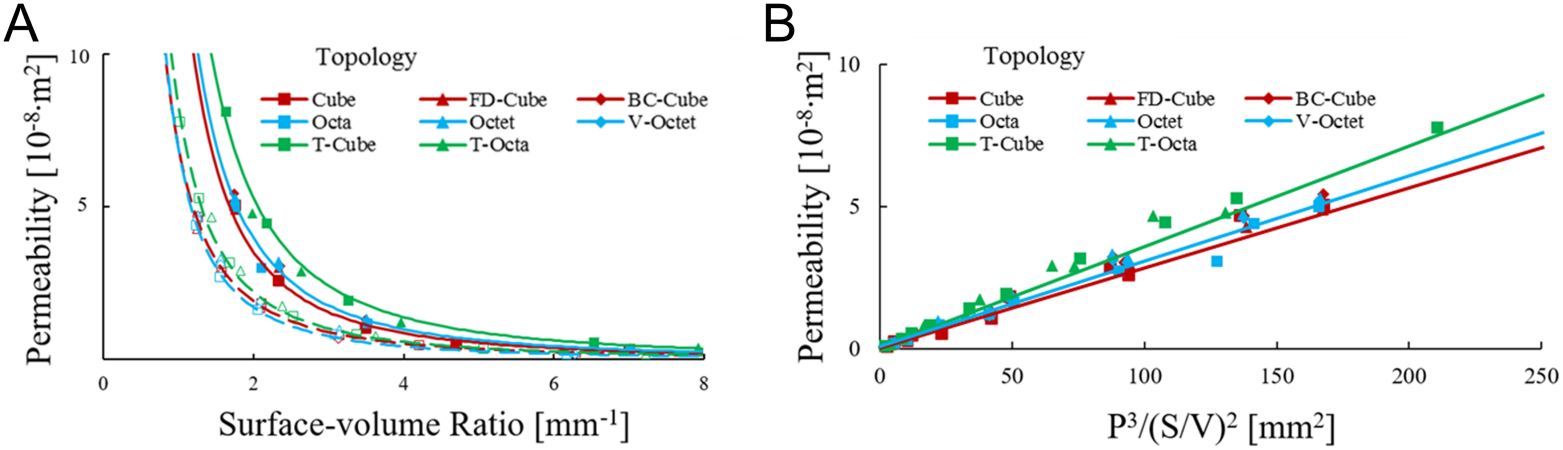
Fluid transport trends. (a) Permeability *k* for each topology when porosity *P* = 0.6 (open symbols; dotted lines) and *P* = 0.8 (closed symbols; solid lines) for surface-volume ratio *S/V*; lines reflect best fits for each unit cell family. (b) The Kozeny-Carmen relation *k* = *K*·*P*^3^ /(*S/V*)^2^ with lines of best fit for each unit cell family.

Permeability *k* was assumed to depend on porosity *P* and surface-volume ratio *S*/*V*, as suggested by the Kozeny-Carmen relation

$$k=K\cdot P^{3}/{\left(S/V\right)}^{2}$$ (5)

where *K* is an empirically fitted coefficient [34–36]. Permeabilities were plotted in Fig 7A by holding porosity at *P* = 0.6 or *P* = 0.8 and tuning beam diameter and unit cell length appropriately for permeability values up to *k* = 1 × 10^−7^*m*^2^, which is on the same order of magnitude as bone [33]. Higher permeability values generally lead to lattices with pore sizes greater than 2000*μm* that are not appropriate for tissue engineering.

Fig 7A shows permeability is higher for topologies with increasing porosity and decreasing surface-volume ratio, and is consistent with the Kozeny-Carmen relation. Power law equations (*k* = *A*·(*S/V*)^−*B*^) were fit to the Cubic (*k* = 0.69 × 10^−7^·(*S/V*)^−1.86^, Octahedron (*k* = 0.68 × 10^−7^·(*S/V*)^−1.99^), and Truncated (*k* = 0.83 × 10^−7^·(*S/V*)^−1.92^) families when *P* = 0.6 and for the Cubic (*k* = 1.38 × 10^−7^·(*S/V*)^−1.99^), Octahedron (*k* = 1.32 × 10^−7^·(*S/V*)^−1.96^), and Truncated (*k* = 2.03 × 10^−7^·(*S/V*)^−1.94^) families when *P* = 0.8 to determine the scaling of surface-volume ratio with permeability; all fits have *R*^2^ ≥ 0.99 and have *B* coefficients suggestive of an inverse square scaling law.

When the same data was assumed to adhere to the Kozeny-Carmen relation *k* = *K*·*P*^3^ /(*S/V*)^2^ in Fig 7B, Kozeny-Carmen coefficients *K* were found with best fit lines for each topology family. The Cubic family was fit with *K* = 2.75 × 10^−7^, the Octahedron family with *K* = 2.77 × 10^−7^, and the Truncated family with *K* = 3.50 × 10^−7^. All fits have *R*^2^ ≥ 0.99 that support the Kozeny-Carmen relation as a predictor for permeability and the high *R*^2^ values suggest that topologies within a given family were grouped appropriately.

### Controlled porosity comparison

The scaling of properties support topology comparisons when a relative density based property (e.g. porosity, elastic modulus, or shear modulus) and pore size are controlled (Fig 2). The relative density defines the ratio of beam diameter to unit cell length for each topology, and defines the porosity, relative elastic modulus, and relative shear modulus for each topology using Fig 6 data. Values for beam diameter and unit cell length parameters were solved for a controlled pore size, and enable the evaluation of surface-volume ratio and permeability using the Kozeny-Carmen relation.

**Fig 8:**
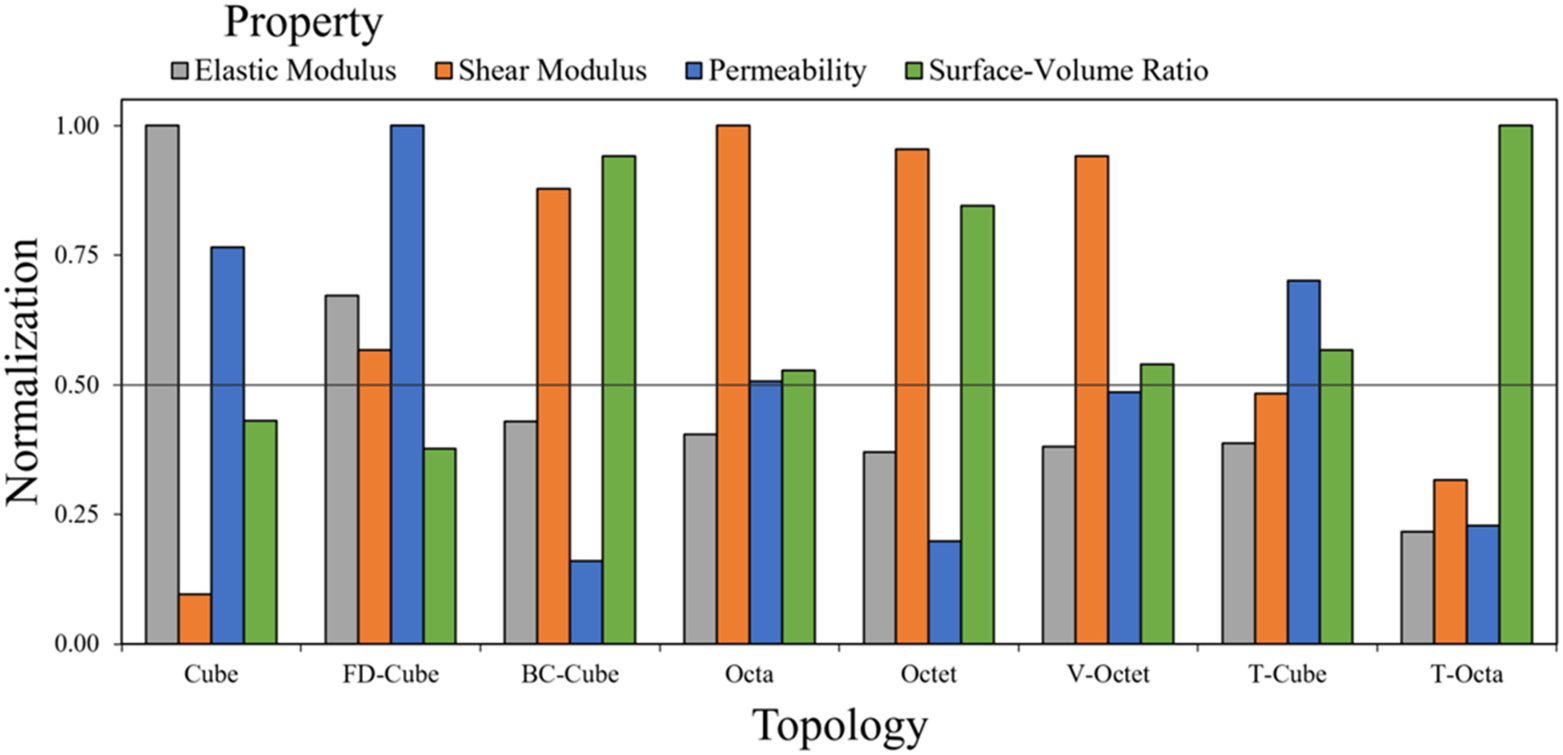
Relative comparison of topologies with fixed porosity and pore size. Properties of lattices with Porosity *P* = 0.8 and pore size *p* = 500μ*m* are normalized to relative elastic modulus *E*_*r*_ = 0.07, relative shear modulus *G*_*r*_ = 0.081, surface-volume ratio *S/V* = 7.0*mm*^−1^ and permeability *k* = 3.1 × 10^−8^*m*^2^.

Topology properties were initially evaluated for a controlled porosity *P* = 0.8 and pore size *p* = 500*μm*. Properties were compared for elastic modulus, shear modulus, surface-volume ratio, and permeability with the highest property value among all topologies for each property used as a normalization factor to facilitate a relative comparison in Fig 8.

Results show the Cube topology has the highest relative elastic modulus (*E*_*r*_ = 0.07), the Octa topology has the highest relative shear modulus (*G*_*r*_ = 0.081), the T-Octa topology has the highest surface-volume ratio (*S/V* = 7.0*mm*^−1^), and the FD-Cube topology has the highest permeability (*k* = 2 × 10^−8^*m*^2^). The only other normalized properties for topologies above 0.7 are the shear modulus for the BC-Cube, Octet, and V-Octet, the permeability for the Cube, and the surface-volume ratio for the BC-Cube and Octet topologies. These findings suggest that topologies have highly specialized properties since only two topologies, the BC-Cube and Octet, have two relative properties above 0.8.

The Cube topology achieves a high elastic modulus suitable for compressive loads in spinal cage applications, but has a normalized shear modulus of only 0.1, with a normalized permeability of 0.78 and normalized surface-volume ratio of 0.43. The FD-Cube has the second highest normalized elastic modulus of 0.67, with a normalized shear modulus of 0.57 and a higher permeability but similar surface-volume ratio to the Cube topology. The BC-Cube, Octa, Octet, and V-Octet topologies all have similar normalized elastic moduli of 0.37 to 0.43. These topologies have comparatively high normalized shear moduli (the lowest being 0.88), except for the T-Cube topology that is 0.48. The BC-Cube and Octet have relatively high normalized surface-volume ratios (0.94 and 0.85) and low normalized permeability (0.16 and 0.20) while the Octa and V-Octet have more balanced values among the two properties (ranging from 0.49–0.54). The T-Cube topology has normalized values for surface-volume ratio and permeability of about 0.54. The T-Octa unit cell has the highest surface-volume ratio but all other normalized values are 0.32 or lower. These results demonstrate the diverse properties attainable by varied topologies, with only the BC-Cube/Octet topologies and Octa/V-Octet topologies sharing highly similar distributions of all properties.

### Controlled elastic modulus comparison

**Fig 9:**
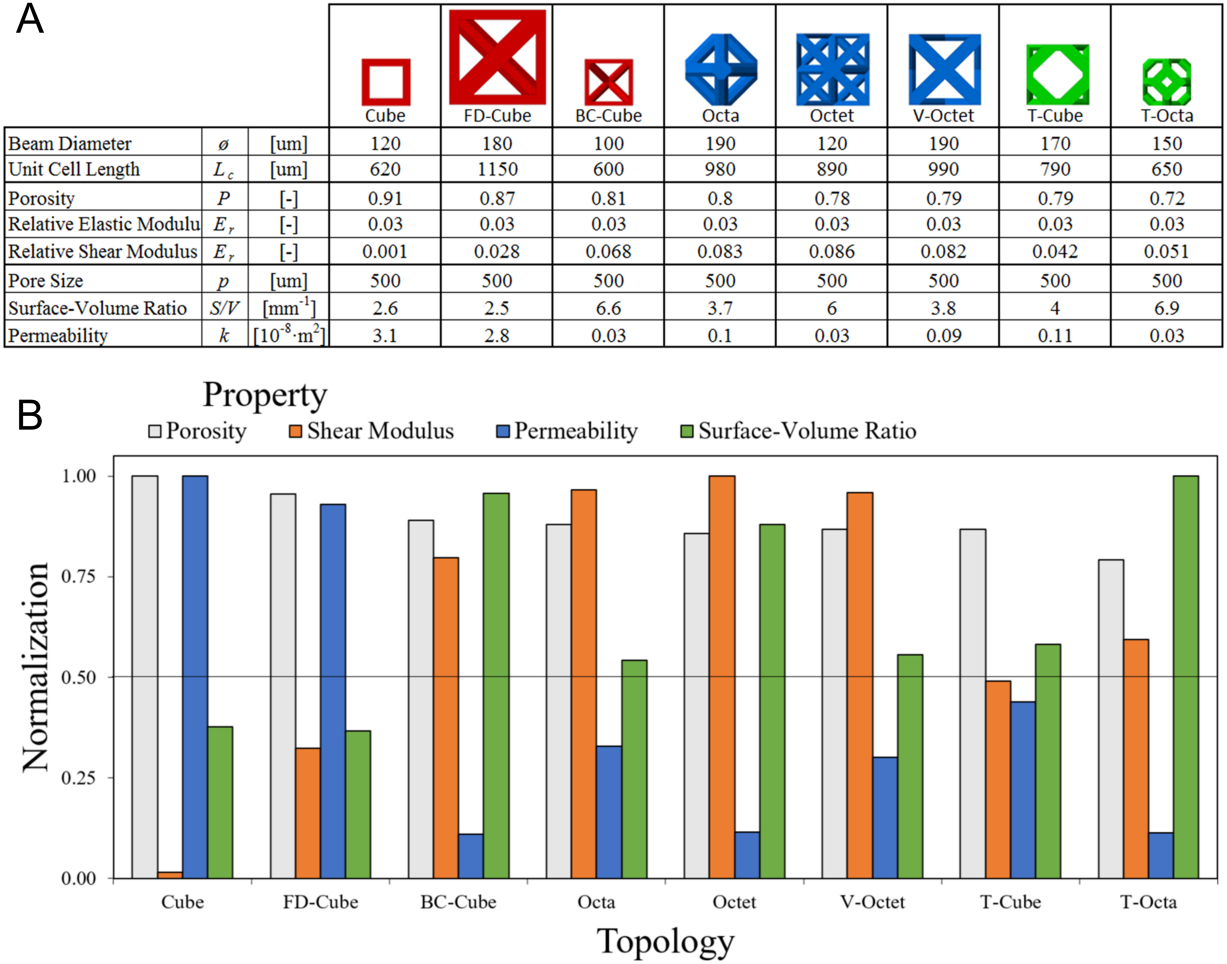
Relative comparison of topologies with fixed elastic modulus and pore size. (a) Lattices were designed with relative elastic modulus *E*_*r*_ = 0.03 and pore size *p* = 500μ*m* and illustrated with a relative scaling. (b) Properties of lattices normalized to porosity *P* = 0.91, relative shear modulus *G*_*r*_ = 0.087, surface-volume ratio *S/V* = 6.9*mm*^−1^ and permeability *k* = 3.1 × 10^−8^*m*^2^.

The porosity controlled comparison has an implicit assumption that alterations in the spinal cage system may compensate for variances among relative elastic moduli for each topology, however, it is also possible to design topologies for a controlled elastic modulus, rather than porosity, to form a basis for comparing relative property differences. A relative elastic modulus of *E*_*r*_ = 0.03 was used since it is within the range of all solved relative elastic moduli in Fig 8 and enables the tuning of lattices with a fixed pore size of *p* = 500*μm* that retain porosities relevant for tissue engineering in Fig 9A.

Fig 9A topologies have beam diameters ranging from 120μm to 190μm and unit cell lengths from 600μm to 1150μm. The FD-Cube is the largest unit cell and the Cube, BC-Cube, and T-Octa are the smallest unit cells. The Cube topology has the highest porosity (*P* = 0.91) and permeability (*k* = 40 × 10^−8^*m*^2^), the Octet topology has the highest shear modulus (*E*_*r*_ = 0.086), and the T-Octa topology has the highest surface-volume ratio (*S/V* = 6.9*mm*^−1^); these properties are normalized and plotted for all topologies in Fig 9B.

All topologies retain a high normalized porosity of 0.79 or higher, with the lowest calculated porosity being *P* = 0.7 for the T-Octa topology while the FD-Cube topology has *P* = 0.87. All other topologies have *P* = 0.8 ± .03. These results contrast with Fig 8 normalized comparisons, since there is a much larger variance in elastic modulus since the lowest normalized elastic modulus in Fig 8 is 0.22 for the T-Octa topology. These results suggest that relative differences among lattices are dependent on controlled property values, possibly due to differing nonlinearities and sensitivities in property relationships.

In contrast to Fig 8, the Fig 9 Cube and FD-Cube topologies have highly similar properties since the FD-Cube has only slightly lower normalized porosity (0.96) and permeability (0.93) that the Cube topology. The normalized shear modulus for the Cube topology (0.02) is much lower in comparison to the FD-Cube topology (0.32). The BC-Cube, Octa, Octet, V-Octet, and T-Cube topologies retain similar normalized values as in Fig 8. The T-Octa topology has a much higher normalized shear modulus (0.27 higher) and slightly lower normalized permeability (0.09 lower) in comparison to Fig 8. The findings suggest the most favorable topology may change based on the specified application and there are a variety of unique trade-offs among lattices to consider for a desired application.

### 3D Printing of lattices with tuned properties

**Fig 10:**
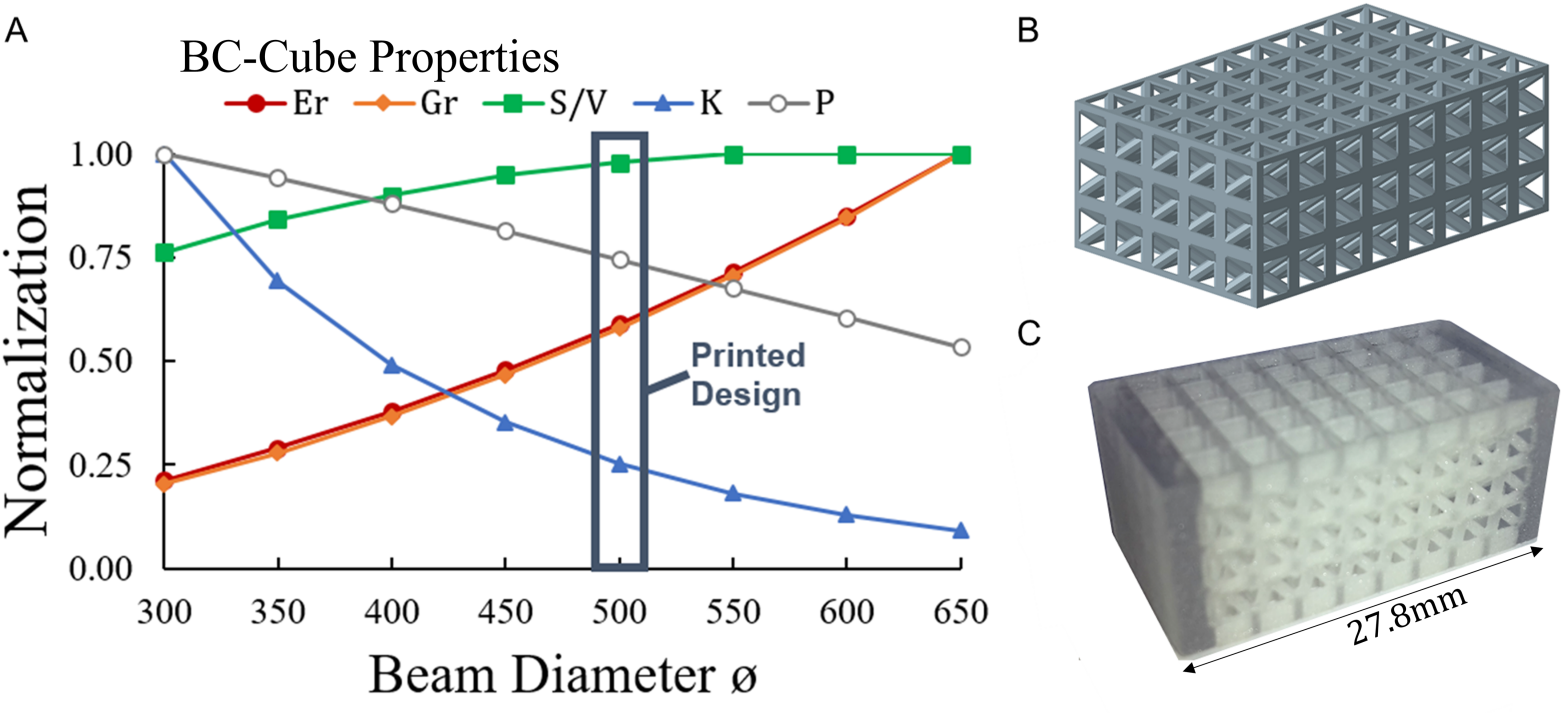
Trade-offs for printed BC-Cube topology for spinal cage applications. (a) BC-Cube lattices were designed with a unit cell size *L*_*c*_ = 2*mm*, while increasing beam diameter for porosities between *P* = 0.45 to to *P* = 0.85; properties were normalized to relative elastic modulus *E*_*r*_ = 0.11, relative shear modulus *G*_*r*_ = 0.24, surface-volume ratio *S/V* = 2.35*mm*^−1^, permeability *k* = 1.25 × 10^−8^*m*^2^, and porosity *P* = 0.85. (b) Designed structure generated by patterning unit cells with beam diameter ø = 500μ*m* and (c) printed design with local reinforcement.

Once a general topology is selected, based on its general relative trade-offs in comparison to other typology types, an in-depth analysis may be conducted to determine a detailed design configuration with properties well-tuned to a particular tissue engineering application. The relative trade-offs of the BC-Cube topology are examined as an example case, with the BC-Cube topology being favorable in Fig 9 as it retains high porosity, shear modulus, and surface-volume ratio for a controlled elastic modulus relative to other topologies. Further optimization of trade-offs for the BC-Cube topology is explored by increasing beam diameter while holding the unit cell size constant for a desired porosity range (Fig 10A).

Comparisons in Fig 10 show that as beam diameter increases, elastic modulus and shear modulus increase, while porosity and permeability decrease. Surface-volume ratio initially increases before reaching a maximum and then decreases. The results demonstrate how lattices of a given topology differ based on specified design parameters, such as beam diameter, and suggest selecting a lattice structure based on trade-offs in achievable properties that may also be manufactured using a suitable 3D printing process. A polyjet process capable of printing lattices with beam diameter of ø = 500μ*m* with a biocompatible polymer [2,56] was selected for demonstrating the fabrication of a BC-Cube lattice with dimensions suitable for the spinal cage application by patterning 3 by 5 by 8 unit cells (Fig 10B).

Prior to printing the designed lattice, local reinforcements were added to the design based on the need for potentially higher structural stiffness in the loading direction when considering spinal loading. The resulting printed structure had dimensions of 12*mm* by 14.9*mm* by 27.8*mm* (Fig 10C). According to Eq 1 the lattice has a pore size of *p* = 1500μ*m*, which is high for bone tissue engineering, but smaller interconnectivity pores between diagonal elements of the lattice could potentially facilitate initial bone growth. Alternatively, a printing process such as selective laser sintering or stereolithography may be utilized to construct lattices with smaller beam diameters or by using the polyjet process while reducing the unit cell size. Local reinforcement of the structure in Fig 10C demonstrates the potential in using 3D printing to produce complex lattices embedded within solid shells, with benefits for bone tissue engineering in spinal fusion applications.

## Discussion

Findings provide unique contributions for identifying and comparing diverse trade-offs for tissue engineering scaffolds constructed with beam-based unit cell libraries [5], with simulation results that suggest all topologies adhere to the Kozeny-Carmen relation for predicting permeability [36]. The design approach is favorable when considering the inherent trade-offs in diverse properties of any local structure [57] and the need to consider such trade-offs in the context of beam-based structures with high mechanical efficiency. The developed method provides a basis for comparing diverse topologies to determine property trends that may be fine-tuned for application specific cases. In the context of bone tissue engineering for the spinal fusion application, the Cube topology provides the highest elastic modulus of all topologies, but the lowest shear modulus which is potentially problematic if other components of the interbody cage system are not robust to shearing. Shearing properties may be improved through adding diagonal elements to the unit cell, as found in the FD-Cube and BC-Cube topologies of the same cubic family. The BC-Cube topology reaches a shearing modulus comparable to topologies with only diagonally aligned loading directions, and includes all members of the octahedron family.

The Octet topology has potentially high performance for bone fusion applications that are not nutrient limited, since it retains a high relative shear modulus and surface-volume ratio albeit relatively low permeability. Topologies of the Truncated family tend to have lower relative properties, however, the T-Octa may be the most favorable topology overall in cases where nutrition and mechanical properties are not limiting since it provides the greatest surface-volume ratio for a given porosity. The T-Cube lattice is potentially favorable since it provides interconnectivity pores of two different sizes that could provide desirable nonlinearities in tissue growth rates. The diverse differences in achievable properties and trade-offs supports the choice in simplifying the design space by investigating specified topology families, since these topologies enable the potential to design a variety of tissue scaffold lattices with tuned properties appropriate for bone tissue engineering.

An optimal set of lattice properties is difficult to determine since scaffold performance is based on complex phenomena informed by property values, and relationships are often non-obvious without further simulations and experiments [63]. For instance, an optimal relative elastic modulus depends on material choice and, in the case of the spinal fusion system, a lattice’s optimal relative elastic modulus value depends on the load carrying capacity of the solid shell of a scaffold and pedicle screws inserted into adjacent vertebra. Additionally, increasing the scaffold permeability is potentially only necessary when nutrient availability limits tissue growth, which suggests a need for modeling the tissue growth rate and nutrient transport [13]. Relative scaffold comparisons could utilize such considerations to provide weighted property assessments that promote favorable balances for tissue growth and nutrient transport. These weightings could inform design decisions between the Octa/V-Octet and BC-Cube/Octet topologies that have similar mechanical properties, but different tunings of surface-volume ratio and permeability.

Quantifications of tissue growth rates could aid in deciding between topologies with highly similar properties but different beam element organizations, such as the BC-Cube and Octet. Relative differences among topologies could also change based on weightings of properties and different normalization approaches [60]. Accurate assessment of trade-offs requires further studies with empirical measurements or more computationally demanding simulations. Further considerations are required to fully assess the performance of scaffolds, such as how the deformation of a scaffold based on its elastic modulus or shear stress from fluid transport stimulate tissue growth [40,44]. If lattices are used for bone tissue engineering in other locations of the body, such as the femur or cranium, optimized design requirements may differ from the spinal fusion case; beam-based titanium scaffolds have been used to support tissue growth for the femur and cranium [3,28]. For the spinal cage, successful fusions have used materials such as tricalcium phosphate and titanium [4]. Lattices with properties tuned for a specific application may be 3D printed as a single part with localized alterations, such as reinforcement to improve stiffness in the direction of principal loading in Fig 10, or with hierarchical features such as removed unit cells that locally improve nutrient transport throughout the lattice [2]. Limitations in 3D printing, such as minimum printable beam diameter, require consideration when tuning application-specific lattices. Further testing is required to determine whether lattices with printed diameters below the 500μm diameter used in Fig 10 are achievable with the polyjet process.

Empirical testing with *in vitro* or *in vivo* tissue growth measurements could provide insights for determining favorable trade-offs among pore size, surface-volume ratio, and permeability [12,13]. Porosity, surface-volume ratio, and pore size properties may be validated by imaging manufactured lattices and comparing results to their corresponding computer generated designs. All topologies from Fig 9 are potentially manufacturable with additive manufacturing technologies such as stereolithography [65]. Element diameter constraints may be introduced to ensure scaffolds are manufacturable with a desired process, such as selective laser sintering that is limited to a minimum beam diameter of 200μm [28]. Mechanical properties of fabricated structures are measurable with standard compression and shear loading tests [5,16,17], while permeability properties are measurable with unidirectional flow chambers [18,66].

Validation for computational predications may commence prior to resource expensive experiments through corroboration with existing experimental data and computational investigations. The results in Fig 6 show Cube topologies have relatively high elastic modulus and low shear modulus in comparison to unit cells with many diagonal beams, and agrees with previous computational approaches using beam-based finite element analysis [29]. The modeled relative elastic modulus of the Octet topology agrees well with studies that have found its relative elastic modulus as *E*_*r*_ ≈ 0.01 when its relative density is about 0.1 [27,67]. Permeability predictions agree with studies that have found permeability on the order of 1 × 10^−8^*m*^2^ for scaffolds with similar porosities and pore sizes tested with unidirectional flow chambers [18,66] and simulated with computational fluid dynamics [36,58]. Surface-volume ratios found for scaffolds are highly similar to those of trabecular bone, that is estimated as 7.25*mm*^−1^ when bone porosity is 0.8 [68]. These validations suggest that although experiments are required to validate computational predictions, the developed approach is suitable for assessing relative properties of designed lattices for spinal fusion cages, or for alternate constraints relevant for diverse bone tissue engineering applications.

## Conclusions

A computational approach was developed for evaluating and comparing beam-based lattices as additively manufactured tissue scaffolds, with spinal fusion selected as an exemplary application for bone tissue engineering. The approach used a library of eight unit cells with varied topologies to generate lattices with controlled properties including porosity, pore size, surface-volume ratio, elastic modulus, shear modulus, and permeability. Finite element analyses were used to quantify scaling relationships of elastic modulus and shear modulus as a function of porosity for each topology. Computational fluid dynamics simulations demonstrated that all topologies adhere to the Kozeny-Carmen relation, such that permeability scales with porosity cubed over surface-volume ratio squared. The developed computational approach provides a basis for evaluating lattice properties analytically or through interpolation from simulated data, at a much faster rate than further finite element analyses or experiments allow.

Lattices were designed with controlled properties of porosity *P* = 0.8 with pore size *p* = 500*μm* and relative elastic modulus *E*_*r*_ = 0.03 with pore size *p* = 500*μm*. Contrasts among the two cases illustrate how relative comparisons of lattice properties depend on application specifications and initial topologies. For instance, the Cube topology achieves the highest elastic modulus for a given porosity while retaining a high permeability, but has a low shear modulus and surface-volume ratio. The Octet topology has a relatively high shear modulus and surface-volume ratio, but low permeability that may be favorable when tissue growth conditions are not limited by scaffold stiffness and nutrient transport. Findings demonstrate relative differences among lattice topologies when key tissue engineering properties are controlled, and provide a basis for tuning lattices with optimized structures suitable for bone tissue engineering, especially in combination with their fabrication using 3D printing.
